# A Genome-Wide Association Study and Polygenic Risk Score Analysis of Posttraumatic Stress Disorder and Metabolic Syndrome in a South African Population

**DOI:** 10.3389/fnins.2021.677800

**Published:** 2021-06-10

**Authors:** Patricia C. Swart, Leigh L. van den Heuvel, Cathryn M. Lewis, Soraya Seedat, Sian M. J. Hemmings

**Affiliations:** ^1^Department of Psychiatry, Faculty of Medicine and Health Sciences, Stellenbosch University, Stellenbosch, South Africa; ^2^South African Medical Research Council, Stellenbosch University Genomics of Brain Disorders Research Unit, Faculty of Medicine and Health Sciences, Stellenbosch University, Cape Town, South Africa; ^3^Social, Genetic and Developmental Psychiatry Centre, King’s College London, London, United Kingdom

**Keywords:** polygenic risk scores, GWAS, PTSD, metabolic syndrome, *PARK2*

## Abstract

Posttraumatic stress disorder (PTSD) is a trauma-related disorder that frequently co-occurs with metabolic syndrome (MetS). MetS is characterized by obesity, dyslipidemia, and insulin resistance. To provide insight into these co-morbidities, we performed a genome-wide association study (GWAS) meta-analysis to identify genetic variants associated with PTSD, and determined if PTSD polygenic risk scores (PRS) could predict PTSD and MetS in a South African mixed-ancestry sample. The GWAS meta-analysis of PTSD participants (*n* = 260) and controls (*n* = 343) revealed no SNPs of genome-wide significance. However, several independent loci, as well as five SNPs in the *PARK2* gene, were suggestively associated with PTSD (*p* < 5 × 10^–6^). PTSD-PRS was associated with PTSD diagnosis (Nagelkerke’s pseudo *R*^2^ = 0.0131, *p* = 0.00786), PTSD symptom severity [as measured by CAPS-5 total score (*R*^2^ = 0.00856, *p* = 0.0367) and PCL-5 score (*R*^2^ = 0.00737, *p* = 0.0353)], and MetS (Nagelkerke’s pseudo *R*^2^ = 0.00969, *p* = 0.0217). These findings suggest an association between PTSD and *PARK2*, corresponding with results from the largest PTSD-GWAS conducted to date. PRS analysis suggests that genetic variants associated with PTSD are also involved in the development of MetS. Overall, the results contribute to a broader goal of increasing diversity in psychiatric genetics.

## Introduction

In South Africa, trauma exposure and a diverse population provide an ideal opportunity to investigate genetic variants associated with posttraumatic stress disorder (PTSD). PTSD is a complex and debilitating trauma-related disorder characterized by intrusive thoughts, increased arousal, avoidance behaviors and negative changes in cognition and mood (American Psychiatric Association, 2013). The South African Stress and Health Study estimated the conditional prevalence of PTSD to be 3.5% (Atwoli et al., 2013), with most South Africans experiencing at least one potentially traumatic event in their life-time (Williams et al., 2007). Current diagnostic measures rely on clinician-based interviews and self-report measures from patients. There is a need to identify individuals at risk for developing PTSD in order to implement early intervention strategies or treatment following trauma exposure. However, individual susceptibility, heterogenous symptoms and varying degrees of trauma severity make predicting, diagnosing, and treating PTSD challenging.

There are several risk factors associated with the development of PTSD, such as level of education (Polimanti et al., 2019; Shalev et al., 2019), the experience of childhood trauma (child neglect and emotional, physical and sexual abuse) (McLaughlin et al., 2017) and prior exposure to community and interpersonal violence (Nöthling et al., 2019; Shalev et al., 2019). In addition, females are more likely to develop PTSD than males (Christiansen and Hansen, 2015). Further, PTSD is frequently associated with symptoms of anxiety and depression (Barbano et al., 2019) and often co-occurs with metabolic syndrome (MetS) (Rosenbaum et al., 2015; Penninx and Lange, 2018). MetS, a risk factor for cardiovascular disease, is characterized by obesity, high blood pressure, dyslipidemia and insulin resistance (Alberti et al., 2009). Individuals with PTSD are at a greater risk for developing MetS compared to age- and sex-matched controls (Rosenbaum et al., 2015; Wolf et al., 2017). The pathophysiology underlying PTSD and its associated co-morbidities remain largely unknown. Although PTSD is conditional upon an extrinsic event(s), there is strong evidence to support the genetic heritability of PTSD (Duncan et al., 2018a). It is, however, unclear how trauma exposure and genetic risk interact to result in PTSD and MetS co-morbidity. Therefore, understanding the genetic architecture of PTSD may help elucidate the physiological mechanisms that lead to the development of the disorder and help identify and treat individuals at risk.

Candidate gene approaches have reported an association between PTSD and variants in genes such as FKBP prolyl isomerase 5 (*FKBP5)*, ADCYAP receptor type I (*ADCYAP1R1)* and C-reactive protein (*CRP)* (reviewed by Daskalakis et al., 2018). Briefly, these genes implicate hypothalamic-pituitary-adrenal axis dysfunction, glucocorticoid dysregulation, and immune system abnormalities in PTSD. These associations are supported by several other studies. For instance, increased hair cortisol levels were observed in South African women with PTSD compared to trauma-exposed controls (Heuvel et al., 2020) and PTSD severity has been associated with higher levels of inflammation (Fonkoue et al., 2020). However, the Psychiatric Genomics Consortium (PGC) (Sullivan et al., 2018) have highlighted the power of genome-wide association studies (GWAS), which simultaneously test the association of common genetic variants across the genome with a phenotype of interest, thereby limiting the usage and bias of candidate gene studies in the field of psychiatric genetics.

The PGC-PTSD Workgroup, an international collaboration investigating the genomics of PTSD, have published two of the largest PTSD GWAS to date (Duncan et al., 2018b; Nievergelt et al., 2019). The PGC-PTSD Freeze 1 dataset (*n* = 4,522 PTSD cases; *n* = 15,548 controls) did not identify any genome-wide significant loci (Duncan et al., 2018b). However, after acquiring additional samples, two independent significant loci were identified in the subset of samples of European ancestry (*n* = 23,212 PTSD cases; *n* = 151,447 controls) and one significant locus was identified in the subset of samples of African ancestry (*n* = 4,363 PTSD cases; *n* = 10,976 controls) in the PGC-PTSD Freeze 2 GWAS (Nievergelt et al., 2019). The genome-wide significant loci and variants in linkage disequilibrium implicated zinc finger DHHC-type palmitoyltransferase 14 (*ZDHHC14*), parkin RBR E3 ubiquitin protein ligase (*PARK2*), kazrin (*KAZN)*, TMEM51 antisense RNA 1 (*TMEM51-AS1*) and zinc finger protein 813 (*ZNF813*) in European samples and long intergenic non-protein coding RNA 2335 (*LINC02335*), microRNA 5007 (*MIR5007*), PCBP2 overlapping transcript (*PCBP2-OT1*), long intergenic non-protein coding RNA 2571 (*LINC02571*) and major histocompatibility complex B (*HLA-B*) in African samples. However, these did not replicate in an independent cohort consisting of European- and African-American participants (Gelernter et al., 2019) and to date, GWAS have yet to identify robust genetic variants associated with PTSD.

This could be due to the fact that PTSD has a complex genomic architecture, with potentially thousands to hundreds of thousands of common single nucleotide polymorphisms (SNPs), each with a small effect size, contributing to the risk for or resilience against toward developing the disorder. Aggregating the effects of these common SNPs, using individual or summary level statistics from GWAS data, can provide insight into the degree to which genetic variants influence the phenotype of interest. For instance, at a population level, the phenotypic variation observed in a cohort of women of European ancestry with PTSD explained by common SNPs (*h*^2^_SNP_), was shown to be ∼ 29% (Duncan et al., 2018b). Interestingly, this was considerably higher than the *h*^2^_SNP_ observed in the corresponding cohort of male participants (Duncan et al., 2018b). Further, the SNP-based heritability estimates for PTSD are comparable to other complex psychiatric disorders such as schizophrenia, bipolar disorder and major depressive disorder (MDD) (Duncan et al., 2018b), highlighting the significant intrinsic contribution of many common genetic variants toward the risk for PTSD.

To assess genetic liability at an individual level, one can employ the use of polygenic risk scores (PRS). A PRS is the sum of all the risk alleles carried by an individual weighted by their effect size for a particular trait (Lewis and Vassos, 2020). In other words, PRS represent the additive effect of thousands to hundreds of thousands of genetic variants as a single measure of genetic risk, on an individual level, toward developing a particular trait. Therefore, PRS has the potential to identify individuals at risk for developing PTSD following trauma exposure and in fact, the genetic risk for PTSD (PTSD-PRS) has been shown to be more predictive of PTSD diagnosis than trauma exposure severity (Waszczuk et al., 2020). The predictive utility of PRS can also explain some of the phenotypic variance in response to trauma exposure, for example, PTSD-PRS explained 4.68% of the variation observed in PTSD onset and 4.35% of PTSD symptom severity in a cohort of war veterans (Misganaw et al., 2019). Delineating individuals based on their PRS for developing PTSD may allow for improved early prevention and treatment interventions to be put in place following trauma or in anticipation of trauma exposure (e.g., emergency service personnel).

Polygenic risk scores can also be used to examine shared genetic risk between traits of interest such as associated co-morbidities (e.g., MetS) and other psychiatric disorders. For example, a modest genetic risk overlap was observed between PTSD and MDD (Duncan et al., 2018b) and MDD-PRS significantly predicted PTSD diagnoses in a cohort of 9/11 responders (Waszczuk et al., 2020) as well as in a civilian Peruvian cohort (Shen et al., 2020). Shared genetic risk strongly suggests joint underlying genetic and physiological mechanisms exist between traits and PRS can therefore also be used to identify shared molecular pathways to provide insight into mechanisms underlying co-morbid disorders.

The studies introduced above have mainly been conducted in cohorts comprising samples of European ancestry, which has left a significant gap in knowledge regarding the genetic contribution to developing PTSD in individuals that are not of European ancestry. For example, due to discrepant allele frequencies between population groups, a risk variant may not be associated with a trait of interest in one population group compared to another. The differences in genomic architecture between various ancestries also impacts the utility of PRS amongst different ancestral groups (Misganaw et al., 2019). This highlights the need for genetic studies consisting of individuals of non-European ancestry in order to increase the diversity of psychiatric genetics research so that genetic-based treatment/intervention strategies and the clinical use of PRS can be beneficial to all population groups.

This study, comprised of a civilian cohort of individuals self-identified as belonging to the South African Colored population group, examined cross-sectionally, aimed (i) to identify genetic variants associated with PTSD by conducting a GWAS meta-analysis (*n* = 343 controls; *n* = 260 PTSD cases), (ii) to determine the predictive utility of PRS for PTSD in this South African cohort, (iii) in order to investigate shared genetic mechanisms between PTSD and MetS. This study represents the first GWAS and PRS analysis of PTSD in a uniquely admixed South African sample.

## Materials and Methods

### Study Population

Participants for this study were recruited between May 2014 and June 2017 as part of the SHARED ROOTS project, conducted in Cape Town, South Africa. The study was approved by Stellenbosch University’s Health Research Ethics Committee (HREC: N13/08/115). All research participants provided written informed consent to take part in the study. Participants were included if they were willing and able to provide informed consent; were 18 years or older; were able to read and write Afrikaans or English; were not pregnant; and were self-identified as being South African Colored. The South African Colored population is a five-way admixed population group, located in the Western Cape Province of South Africa (Uren et al., 2016, 2020). Participants were excluded if they had any major psychiatric disorder (e.g., severe psychotic or bipolar disorder), or any neurological disorder.

### Demographic and Clinical Assessment

Sociodemographic data, such as gender, ethnicity, age, education, employment, income, and marital status, were ascertained using a demographic questionnaire. Clinicians diagnosed participants with PTSD, and assessed PTSD symptom severity over the previous month, using the Clinician-Administered Posttraumatic Stress Disorder Scale (CAPS-5) for Diagnostic and Statistical Manual of Mental Disorders, 5th edition (DSM-5) (Weathers et al., 2015). In addition, PTSD symptom severity (range 0–80) was also determined using the PTSD Checklist for DSM-5 (PCL-5), with a cut-off point between 31 and 33 suggestive of probable PTSD diagnoses (Blevins et al., 2015). Where possible, control participants were matched on ethnicity, age, gender and trauma-exposure, based on the DSM-5 criteria.

Metabolic syndrome screening was conducted using the WHO STEPS instrument (WHO, 2008). Blood pressure, heart rate, height, weight, and waist circumference were measured. In addition, venous blood samples were drawn after an overnight fast (of at least 8 h), to assess levels of fasting blood glucose, triglyceride and high-density lipoprotein cholesterol (HDL-C). Participants were diagnosed with MetS if they were found to have three out of five of the following harmonized JIS criteria: (i) raised waist circumference (≥90 cm); (ii) raised triglycerides (>1.7 mmol/l); (iii) low HDL-C (men < 1.0 mmol/l, women < 1.3 mmol/l); (iv) raised blood pressure ≥130/85 mmHg or on hypertension treatment; and (v) raised fasting glucose ≥5.6 mmol/l or on diabetes treatment. These criteria and cut-off values represent a widely used consensus definition of MetS derived from a meeting of several health organizations (Alberti et al., 2009). In addition, body mass index (BMI), an alternative clinical measure of adiposity to asses MetS (Gurka et al., 2018), was used as a continuous measure of cardiovascular disease risk in the PRS models.

### Genotype Quality Control and Imputation

DNA from participants with PTSD and controls was extracted from blood samples using the Gentra Puregene Blood Kit (Qiagen), according to the manufacture’s protocol, in the Neuropsychiatric Genetics Laboratory at Stellenbosch University, South Africa. One-hundred and sixty-three of the controls and 164 participants with PTSD were genotyped using the Infinium Multi-ethnic Global Array (MEGA, Illumina) (Wave 1) and 221 controls and 117 participants with PTSD were genotyped using the Infinium Global Screening Array (GSA, Illumina) (Wave 2). All genotyping was conducted at the Broad Institute (Cambridge, MA, United States) in collaboration with the Psychiatric Genomics Consortium and Cohen Veteran Society. Note, due to the complex genomic architecture of the study population, a 5-way admixed population, the samples genotyped through this collaboration were not included in the PGC-PTSD Freeze 2 GWAS meta-analysis and therefore, the discovery and target datasets used for the PRS analysis (see section “Polygenic Risk Score Analysis”) are independent.

Genotyping quality control procedures were performed separately on the genotype data obtained from Wave 1 and Wave 2 samples using PLINK 1.9 (Chang et al., 2015). Briefly, variants were restricted to SNPs located on chromosomes 1–22 and were excluded if they had a minor allele frequency (MAF) < 1%, a missingness rate >3%; a significantly different call rate between cases and controls; or failed Hardy-Weinberg Equilibrium in control samples (*p*-value < 1 × 10^–6^) (Schurz et al., 2019). Individual samples were removed if they were found to have excessive missingness, a mean heterozygosity rate greater than three times the standard deviation, and mismatching sex information (Marees et al., 2018). Related samples were identified using the pairwise identity-by-descent function in PLINK 1.9 and PI_HAT > 0.2 (Anderson et al., 2010; Marees et al., 2018). Thirty-three and 31 samples were removed due to relatedness in Wave 1 and Wave 2, respectively.

Genotype principal component analysis (PCA) of PTSD cases and controls was performed using SMARTPCA (Patterson et al., 2006; Price et al., 2006), after restricting SNPs to variants with rsIDs only; removing SNPs in linkage disequilibrium (*–indep-pairwise* 50 5 0.2); and removing related individuals (PI_HAT > 0.2) (Anderson et al., 2010; Marees et al., 2018). In addition, SMARTPCA was used to show how the South African Colored population fits in among global ancestral groups. This was done by merging these genotype data with 1000 Genomes Project (1KGP) Phase3 data (Auton et al., 2015).

In order to increase coverage, genotype imputation was conducted using SHAPEIT2 (Delaneau et al., 2011) and the positional Burrows-Wheeler transform (PBWT) (Durbin, 2014) via the Sanger Imputation Server (McCarthy et al., 2016) and the African Genome Resource (AGR) reference panel^1^. Supplementary Figure 1 illustrates that imputation with the AGR reference panel produced higher median quality scores compared to other available reference panels. The AGR has previously been shown to be the most suitable, publicly available imputation panel for the South African Colored population (Schurz et al., 2019). Following imputation, SNPs with an INFO Score > 0.8, a MAF > 1% and a missingness rate <3% were retained for further analysis.

### GWAS Meta-Analysis

Genome-wide association analysis was conducted following quality control and imputation within Wave 1 (controls, *n* = 141; PTSD, *n* = 153) and Wave 2 (controls, *n* = 202; PTSD, *n* = 107). Logistic regression was performed to test the association between genotype and PTSD diagnosis with the first five PCs included as covariates (Supplementary Figure 2). Sex was not included as a covariate because the number of male and female participants did not differ between the case and control groups (χ^2^, *p* > 0.05, Table 1). However, age was included as an additional covariate in the Wave 2 GWAS because control subjects were significantly older than participants with PTSD in (Wilcoxon, *p* < 0.001, Table 1). A fixed-effects meta-analysis was then conducted across Wave 1 and Wave 2 using *p*-values and direction of effect, weighted by sample size, in METAL (Willer et al., 2010). A *p*-value below 5 × 10^–8^ was considered statistically significant, according to Bonferroni multiple test correction for one million SNPs. A SNP with a *p*-value below 5 × 10^–6^ was considered to be suggestively associated with PTSD.

**TABLE 1:** Descriptive characteristics of Wave 1 and Wave 2 participants.

	Wave 1			Wave 2		
		
	Controls (*n* = 141)	PTSD (*n* = 153)	*p*-value	Controls (*n* = 202)	PTSD (*n* = 107)	*p*-value
Age, years	42.0 (IQR = 21.7)	40.6 (IQR = 17.6)	0.232	50.8 (IQR = 22.1)	43.9 (IQR = 18.9)	<0.001***
Sex (Female)	75.1% (*n* = 106)	74.5% (*n* = 114)	1	59.9% (*n* = 121)	69.1% (*n* = 74)	1
MetS (Yes)	27.6% (*n* = 39)	28.7% (*n* = 44)	0.937	34.6% (*n* = 70)	31.8% (*n* = 34)	0.702
BMI	27.3 (IQR = 8.6)	28.5 (IQR = 10.5)	0.724	28.4 (IQR = 10.3)	28.7 (IQR = 10.7)	0.722
CAPS-5 total score	4.0 (IQR = 11.0)	37.0 (IQR = 13.0)	<0.001***	6.0 (IQR = 11.0)	34.0 (IQR = 16.0)	<0.001***
PLC-5 total score	9.0 (IQR = 17.2)	53.0 (IQR = 19.5)	<0.001***	10.5 (IQR = 24.8)	48.0 (IQR = 24.0)	<0.001***

MetS, metabolic syndrome; BMI, body mass-index; CAPS-5, clinician-administered PTSD scale for DSM-5; PCL-5, PTSD checklist for DSM-5; *** indicates a p-value less than 0.001.

### Functional Annotation

The GWAS meta-analysis output was annotated according to the human genome build GRCh37 (hg19) using the default settings of the SNP2GENE function in FUnctional Mapping and Annotation (FUMA), a web-based tool (Watanabe et al., 2017). SNPs were considered independent from each other at a default *r*^2^ value of 0.6.

### Polygenic Risk Score Analysis

PRSice version 2.3.1 (Choi and O’Reilly, 2019) was used to calculate PTSD polygenic risk scores (PTSD-PRS) in Wave 1 and Wave 2 participants using summary statistics from the PGC-PTSD Freeze 2 GWAS dataset (Nievergelt et al., 2019), available at https://www.med.unc.edu/pgc/download-results/ptsd/, to assess whether PTSD-PRS was associated with PTSD status and/or PTSD symptom severity, as measured by CAPS-5 and PCL-5, MetS diagnosis (having at least three out of five criteria) and BMI. PTSD-PRS analysis was conducted using the overall PGC-PTSD Freeze-2 GWAS summary statistics (ALL), as well as data from European- (EURO) and African American- (AfAM) ancestry subsets in order to determine which discovery dataset is best suited for this study population.

In R (R Core Team, 2020), PTSD-PRS, at eight *p*-value cut-off thresholds (*P*_T1_ = 0.001, *P*_T2_ = 0.05, *P*_T3_ = 0.1, *P*_T4_ = 0.2, *P*_T5_ = 0.3, *P*_T6_ = 0.4, *P*_T7_ = 0.5 and *P*_T8_ = 1), were regressed on the first five principal components in both Wave 1 and Wave 2 participants. The phenotype of interest was then regressed on the standardized, combined residuals. The variance explained by the regression model was denoted by Nagelkerke’s pseudo *R*^2^ value if the outcome variable was binary (PTSD case/control status and MetS diagnosis) or *R*^2^ if the outcome variable was continuous (PTSD symptom severity score (CAPS-5 and PCL-5) and BMI) (Choi and O’Reilly, 2019). A *p*-value of less than 0.00625 was determined as significant according to Bonferroni multiple test correction (*p* < 0.05/8 *p*-value thresholds).

### Statistical Analysis

In order to determine differences between PTSD cases and controls, within each Wave, non-parametric data (age, CAPS-5 score, and PCL-5 score) were analyzed using a Wilcoxon test and represented by the median and inter-quartile range (IQR). Differences in categorical variables (sex and MetS diagnosis) between PTSD cases and controls were analyzed using a Chi-squared (χ^2^) test. Statistical analysis was conducted in R 4.0.2 or higher (R Core Team, 2020). The package *ggplot2* (Wickham, 2016) was used to generate the figures in R.

## Results

### Study Population Description

One-hundred and forty-one controls and 153 participants with PTSD remained after 33 samples were excluded from the Wave 1 dataset due to relatedness estimates determined during genotype data quality control procedures. Similarly, 31 samples were excluded from the Wave 2 dataset due to relatedness which left 202 controls and 107 participants with PTSD for association analysis.

Within the Wave 1 dataset, controls were of similar age to PTSD participants. Participants with PTSD did not differ from controls on BMI. Both PTSD and control groups had a similar proportion of female participants as well as a similar number of participants with a MetS diagnosis (Table 1). Wave 2 controls were significantly older than participants with PTSD (Wilcoxon, *p* < 0.001, Table 1). There were no significant differences in BMI, sex and MetS frequency between PTSD cases and controls in Wave 2.

**FIGURE 1:**
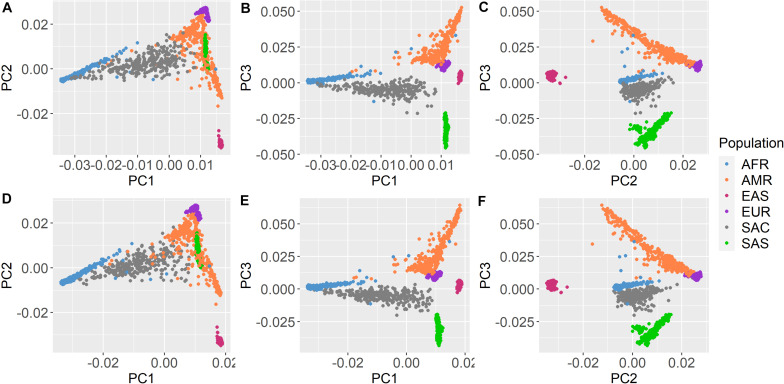
Principal component analysis with 1000 Genomes Project Phase 3 data. Shared Roots **(A–C)** Wave 1 and **(D–F)** Wave 2 participants and 1000 Genomes Phase 3 samples plotted together based on principal components (PC1–3) from overlapping SNP data. Shared Roots participants (gray) cluster separately from the other population groups. AFR, African ancestry; AMR, Admixed American ancestry; EAS, East Asian ancestry; EUR, European ancestry; SAS, South Asian ancestry; and SAC, South African Colored (Shared Roots).

All participants were self-identified as belonging to the South African Colored population group. The genetic distribution of this population within the global ancestry groups is illustrated by Figure 1. The South African Colored population group cluster separately from the other global population groups based on their genetic data. Supplementary Figure 2 illustrates that participants with PTSD and controls were evenly distributed across the principal components.

### GWAS Meta-Analysis

**FIGURE 2:**
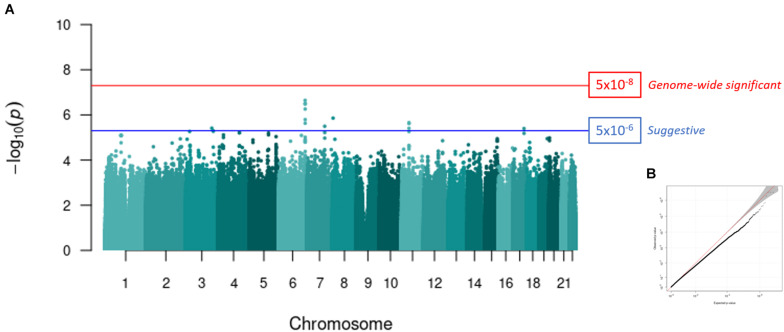
GWAS meta-analysis of PTSD in a South African cohort. **(A)** A Manhattan plot representing the GWAS meta-analysis (controls, *n* = 343; PTSD cases, *n* = 260) of Wave 1 and Wave 2. The red line indicates the genome-wide significance threshold after Bonferroni correction (*p* < 5 × 10^–8^) and the blue line represents a suggestive significance threshold (*p* < 5 × 10^–6^). Seven independent loci reached a suggestive level of genome-wide significance. SNP rs2315551 on chromosome six had the smallest *p*-value (*p* = 2.262 × 10^–^*^7^*). **(B)** A Q-Q plot of the expected vs. observed *p*-values obtained from the meta-analysis illustrates the absence of confounding population structure (genomic inflation, λ = 0.992).

Six-hundred and three samples were included in the meta-analysis. No SNPs reached genome wide significance (*p* < 5 × 10^–8^, Figure 2A). However, seven independent loci reached a suggestive level of significance (*p* < 5 × 10^–6^, Figure 2A and Table 2): rs2315551 (chr6, *p* = 2.262 × 10^–7^), rs9458519 (chr6, *p* = 3.212 × 10^–7^), rs58910976 (chr8, *p* = 1.388 × 10^–6^), rs2084346 (chr11, *p* = 2.202 × 10^–6^), rs1419748 (chr7, *p* = 3.178 × 10^–6^), rs6791269 (chr3, *p* = 3.884 × 10^–6^) and rs77235638 (chr17, *p* = 4.006 × 10^–7^), with a consistent direction of effect between the two datasets. A quantile-quantile (Q-Q) plot indicates the absence of confounding population structure (genomic inflation, λ = 0.992, Figure 2B). However, the Q-Q plot appears visibly deflated. Supplementary Figure 4 illustrates an investigation into the source of this deflation by plotting the expected vs. observed *p*-values per MAF bin.

**TABLE 2:** Seven independent lead SNPs (*p* < 5 × 10^–6^) were identified by FUMA in 603 samples.

Variant	Mapped Gene	Region	Chr	Position	A1	A2	MAF *Wave 1*	MAF *Wave 2*	*Z*-score	Beta^a^	*p*-value	Direction of effect	No. SNPs in LD (*r*^2^ > 0.6)
rs2315551	*PARK2*	Intronic	6	162134994	T	C	0.4354	0.4191	5.176	0.2941	2.262 × 10^–7^	++	1
rs9458519	*PARK2*	Intronic	6	162739309	T	G	0.3537	0.3317	−5.111	−0.3013	3.212 × 10^–7^	–	12
rs58910976	*CSMD1*	Intronic	8	3552673	A	G	0.2551	0.2832	4.827	0.3129	1.388 × 10^–6^	++	3
rs2084346	Uncharacterized	ncRNA-intronic	11	45751081	A	T	0.2874	0.2492	−4.734	−0.2957	2.202 × 10^–6^	–	5
rs1419748	*DOCK4*	Intronic	7	111374061	T	G	0.4983	0.4693	4.659	0.2636	3.178 × 10^–6^	++	2
rs6791269	*TRIM59-IFT80*	Intronic	3	159954631	T	C	0.1531	0.1052	−4.617	−0.3628	3.884 × 10^–6^	–	1
rs77235638	*ABCA8*	Intergenic	17	66957323	T	C	0.0561	0.0518	−4.611	−0.5670	4.006 × 10^–7^	–	3

*Suggestive significance level, *p* < 5 × 10^–6^. Chr, chromosome; A1, minor allele; MAF, minor allele frequency; LD, linkage disequilibrium. ^a^Estimated Beta calculated from *Z*-score.*

The web-based tool FUMA, annotated the seven independent lead SNPs of suggestive significance, to five genes: *PARK2*, CUB and Sushi multiple domains 1 (*CSMD1)*, dedicator of cytokinesis (*DOCK4)*, chromosome 3 open reading frame 80 (*C3orf80*), and ATP binding cassette subfamily A member 8 (*ABCA8)* (Table 2). Gene-based analysis further revealed *PARK2* as a genome-wide significant gene (*p* = 2.6473 × 10^–6^) out of a total of 18,861 protein-coding genes. Gene-set analysis did not reveal any pathways significantly associated with PTSD.

### Polygenic Risk Score Analysis

**FIGURE 3:**
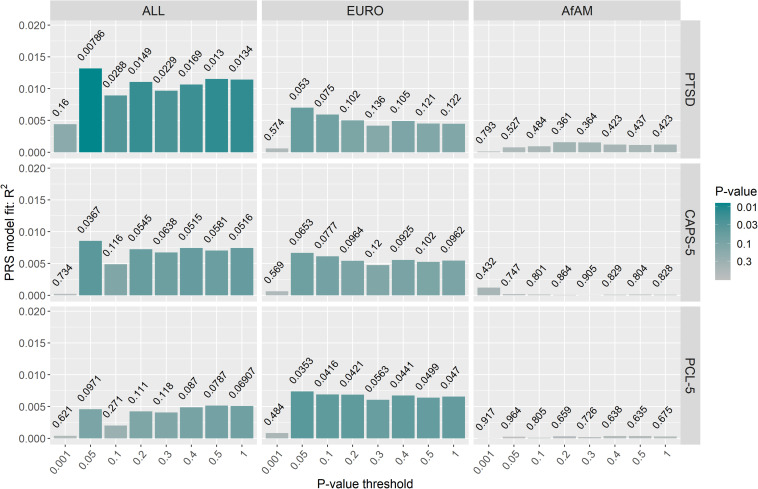
PTSD-PRS prediction for PTSD-related phenotypes (PTSD case/control status, CAPS-5 score and PCL-5 score). The lowest *p*-value was obtained at *P*_T2–ALL_ = 0.05 using the overall (ALL) PGC-PTSD Freeze 2 GWAS summary statistics (*p* = 0.00786). This explained 1.31% of the PTSD case/control phenotypic variation. However, PTSD-PRS, calculated using the PGC-PTSD Freeze 2 GWAS summary statistics, was not predictive of PTSD or PTSD symptom severity after Bonferroni correction for multiple testing (*p* > 0.00625). CAPS-5, clinician-administered PTSD symptom for DSM-5; PCL-5, PTSD checklist for DSM-5; ALL, overall PGC-PTSD Freeze 2 data; EURO, European ancestry; AfAM, African American ancestry.

**FIGURE 4:**
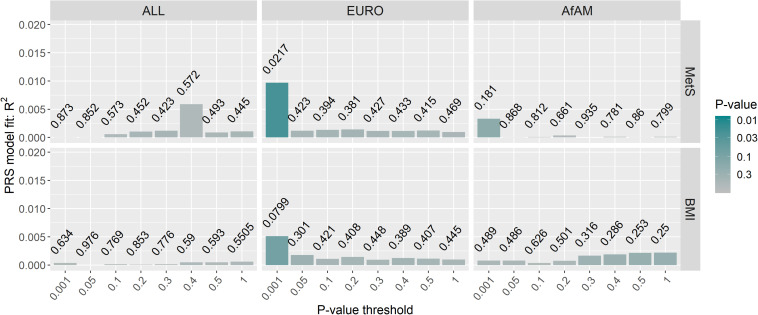
PTSD-PRS prediction for metabolic syndrome-related phenotypes. The lowest *p*-value was obtained at *P*_T1–EURO_ = 0.001 using the European (EURO) PGC-PTSD Freeze 2 GWAS summary statistics (*p* = 0.0217). This explained 0.97% of the variation in MetS diagnosis. Results were not significant after Bonferroni correction for multiple testing (*p* > 0.00625). MetS, metabolic syndrome diagnosis; BMI, body mass index; ALL, overall PGC-PTSD Freeze 2 data; EURO, European ancestry; AfAM, African American ancestry.

Posttraumatic stress disorder-polygenic risk scores were calculated in Wave 1 and Wave 2 participants (controls *n* = 343; PTSD, *n* = 260) using the PGC-PTSD Freeze 2 GWAS summary statistics (ALL, overall; EURO, European-ancestry; and AfAM, African American-ancestry) to assess whether PTSD-PRS was associated with PTSD status, PTSD symptom severity and MetS-related phenotypes in this South African sample. Regression analysis obtained the lowest *p*-value at *P*_T2–ALL_ = 0.05 using the overall (ALL) PGC-PTSD Freeze 2 GWAS summary statistics (*p* = 0.00786). This explained 1.31% of the PTSD case/control phenotypic variation, as measured by Nagelkerke’s pseudo *R*^2^ value (Figure 3). Likewise, regression analysis for CAPS-5 total scores obtained a *p*-value of 0.036 at *P*_T2–ALL_ = 0.05, which explained 0.86% of the phenotypic variation in CAPS-5 total scores (Figure 3). PTSD-PRS was associated with PCL-5 score (*P*_T2–EURO_ = 0.05, *R*^2^ = 0.00737, *p* = 0.0353, Figure 3); and MetS diagnosis (*P*_T1–EURO_ = 0.001, Nagelkerke’s pseudo *R*^2^ = 0.00969, *p* = 0.0217, Figure 4) when using the European PGC-PTSD Freeze 2 GWAS summary statistics. However, these results were not significant after Bonferroni correction for multiple testing (Bonferroni corrected *p*-value = 0.00625). Further, PTSD-PRS calculated using the African American (AfAM) PGC-PTSD Freeze 2 GWAS summary statistics (Figures 3, 4) was not associated with any of the phenotypes of interest (PTSD diagnosis, PTSD symptom severity, MetS diagnosis, and BMI).

## Discussion

To the best of our knowledge this is the first GWAS and PRS analysis of PTSD in the South African population. Despite the modest sample size (*n* = 603), the results of the GWAS meta-analysis suggest that variants in *PARK2* may be associated with the development of PTSD, which is in agreement with the largest PTSD-GWAS meta-analysis conducted to date (Nievergelt et al., 2019). In addition, PRS indicate a possible role of PTSD-associated genetic risk in PTSD-MetS comorbidity, despite the discovery datasets being mostly made up of samples of European ancestry.

The seven independent lead SNPs of suggestive evidence for association (*p*-value below 5 × 10^–6^) in the GWAS meta-analysis, implicated five genes in the development of PTSD (*PARK2*, *CSMD1*, *DOCK4*, *C3orf80*, and *ABCA8*). Briefly, variants in *CSMD1*, encoding the CUB and Sushi multiple domains 1 protein, have previously been associated with PTSD following combat exposure in participants from the Marine Resiliency Study, with 85% of the participants being of European ancestry (Nievergelt et al., 2015). Further, *CSMD1* has also been implicated in schizophrenia (The Schizophrenia Psychiatric Genome-Wide Association Study Consortium, 2011), bipolar disorder (Woo et al., 2017) and cognitive function (Norwegian/Scandinavian cohort) (Athanasiu et al., 2017). *CSMD1* plays a role in the complement system which is involved in the immune system but also in synaptic pruning, a crucial mechanism in neurodevelopment and cognitive processes (Stephan et al., 2012). *DOCK4* has been implicated in schizophrenia and autism spectrum disorder (Koomar and Michaelson, 2020). *DOCK4* regulates adherens junctions between cells and plays a role in dendritic growth, neurodevelopmental processes as well as neurotransmission (Ueda et al., 2008).

Of significance interest is the suggestive association between variants in the *PARK2* gene (also known as *PRKN*) and PTSD in this South African cohort, because *PARK2* has previously been associated with PTSD in the largest PTSD-GWAS meta-analysis conducted to date (Nievergelt et al., 2019). *PARK2* encodes parkin, an E3 ubiquitin ligase involved in many cellular processes throughout the human body. For reasons yet to be fully understood, although evidence points to mitochondrial dysfunction, loss of function associated with variants within *PARK2* results in the degeneration of dopaminergic neurons, specifically (Shaltouki et al., 2015; Sassone et al., 2017; Noda et al., 2020). Hence, *PARK2* genetic variation has been found to be associated with decreased levels of dopamine and the development of Parkinson’s disease (PD) (Blauwendraat et al., 2020). It is notable that war veterans with PTSD are found to have increased risk of developing PD compared to matched-controls (White et al., 2020). Moreover, symptoms of PTSD, such as intrusive (re-experiencing) thoughts, avoidance behaviors, hyperarousal and negative alterations in cognition and mood can be explained by a deficit in dopaminergic signaling in the brain (reviewed by Torrisi et al., 2019) and may unite various PTSD comorbidities, such as MDD (Ney et al., 2021). Therefore, it is plausible that genetic variation in *PARK2* contributes to deficits in dopaminergic signaling observed in the pathophysiology of PTSD. Importantly, even though results from this study are in line with existing literature, before conclusions can be drawn, further work is needed to validate these findings.

Results from the PRS analysis demonstrated that PTSD-PRS constructed from the overall PGC-PTSD Freeze 2 GWAS summary statistics was associated with a diagnosis of PTSD in this South African population. Further, PTSD-PRS was associated with MetS diagnosis, but not BMI, when using a subset of PGC-PTSD Freeze 2 that only consisted of data from individuals of European-ancestry. This finding supports the well-documented association between PTSD and MetS, suggesting that the genetic variants involved in the development of PTSD also play a role in the development of MetS. This may provide insight into mechanisms underlying PTSD-MetS comorbidity.

Results from the PRS analysis need to be considered with caution because the PTSD-PRS only explains a small proportion of the observed phenotypic variation (<2%) in PTSD and MetS and, further, the significant findings do not withstand correction for multiple testing. This may be because the use of PRS is less applicable in non-European populations due to the current lack of large available GWAS summary statistics from cohorts of non-European or admixed ancestry that can be used as discovery data (Vassos et al., 2017). PRS analysis performs better when the discovery and target datasets are derived from the same ancestral population (Misganaw et al., 2019). For example, using the PGC-PTSD Freeze 1 summary statistics, the PTSD-PRS explained 4.68% of the phenotypic variation in a cohort of European veterans, but the predictive ability of the same PRS was minimal in a sub-set of veterans of African ancestry (Misganaw et al., 2019).

Currently, the PGC-PTSD Freeze 2 GWAS overall summary statistics are the largest discovery dataset available to calculate PTSD-PRS (*n* = 206,655 participants). However, it may not be the most optimal discovery dataset to calculate PTSD-PRS in individuals belonging to the South African Colored population, even with the option to stratify the discovery dataset by ancestry because the resultant subsets are much smaller in sample size. This may explain why the PTSD-PRS calculated using the African American (*n* ∼ 15,000 participants) subset of the PGC-PTSD Freeze 2 GWAS was not predictive of any of the phenotypes of interest.

In addition, ancestral make-up between the discovery and target datasets is suggested to be a major PRS performance indicator (Misganaw et al., 2019). Therefore, utilizing the overall summary statistics probably produced the lowest *p*-value because the dataset consists of genetic data from multiple ancestry groups, similar to the varying ancestral contributions to the South African Colored genome (Uren et al., 2016, 2020). However, this does not explain why we only observed an association between PTSD-PRS and MetS diagnosis when using the European subset of PGC-PTSD Freeze 2 summary statistics. As the psychiatric genetics field slowly diversifies its studies, or develops alternative techniques better suited for admixed populations and populations of non-European ancestry, PRS analysis and predictive performance in non-European cohorts should improve, thereby strengthening the statistical as well as clinical applicability of PRS across the globe.

While this study has yielded important preliminary findings, there are limitations that deserve mention. First, this study was considerably underpowered, with a total of *n* = 603 participants included in the final meta-analysis, and neither GWAS nor PRS results withstood correction for multiple testing. Results from this study require replication in more powerful datasets. Second, statistical genetic tools and the GWAS and PRS approaches used in the current study may not be appropriate for the complex genetic architecture of the South African Colored population, corroborating the call for analytical pipelines that are designed specifically to handle genomic data of complex admixed populations as well as the need to increase the diversity of the psychiatric genetics field to facilitate accuracy and clinical application of underlying genetic risk measures. Lastly, with larger samples, future work should consider analyzing PTSD and MetS by symptom clusters (i.e., applying a dimensional approach) rather than categorically by diagnosis in order to provide insight into the mechanisms underlying the heterogenous symptoms characteristic of each disorder.

In summary, results from this study provide supporting evidence for the role of genetic variation in *PARK2* in the development of PTSD. Results from the PRS analysis suggest that genetic risk variants associated with PTSD are also involved in the etiology of MetS thereby providing insight into PTSD-MetS comorbidity. Examining the genomic data in conjunction with additional omics data such as gene expression data and epigenetic data will facilitate increased confidence in the findings. Nonetheless, the study and findings contribute to the broader goal of increasing diversity in psychiatric genetics.

## Data Availability Statement

The datasets presented in this article are not readily available due to ethical and legal restrictions. Requests to access the datasets should be directed to SH (smjh@sun.ac.za). The authors are open to collaborating and sharing data within the limits of ethical review restrictions and data transfer policies of Stellenbosch University.

## Ethics Statement

The studies involving human participants were reviewed and approved by the Stellenbosch University’s Health Research Ethics Committee (HREC: N13/08/115). The patients/participants provided their written informed consent to participate in this study.

## Author Contributions

PS conducted the data analysis and drafted the manuscript. LH acquired the clinical data and managed the project database. CL assisted with and critically assessed the data analysis. SS and SH acquired the genotype data via collaboration with the Psychiatric Genomics Consortium. All authors contributed important intellectual content to the interpretation of the results and took full responsibility for the accuracy and integrity of the work.

## Conflict of Interest

The authors declare that the research was conducted in the absence of any commercial or financial relationships that could be construed as a potential conflict of interest.

## Footnotes

1 https://www.apcdr.org/

## References

[B1] Alberti K. G. M. M. Eckel R. H. Grundy S. M. Zimmet P. Z. Cleeman J. I. Donato K. A. (2009). Harmonizing the metabolic syndrome: a joint interim statement of the International Diabetes Federation Task Force on Epidemiology and Prevention; National Heart, Lung, and Blood Institute; American Heart Association; World Heart Federation; International Atherosclerosis Society; and International Association for the Study of Obesity. *Circulation* 120 1640–1645. 10.1161/CIRCULATIONAHA.109.192644 19805654

[B100] American Psychiatric Association (2013). *Diagnostic and Statistical Manual of Mental Disorders*, 5th Edn. Washington, DC: American Psychiatric Association. 10.1176/appi.books.9780890425596

[B2] Anderson C. A. Pettersson F. H. Clarke G. M. Cardon L. R. Morris A. P. Zondervan K. T. (2010). Data quality control in genetic case-control association studies. *Nat. Protoc.* 5 1564–1573. 10.1038/nprot.2010.116 21085122 PMC3025522

[B3] Athanasiu L. Giddaluru S. Fernandes C. Christoforou A. Reinvang I. Lundervold A. J. (2017). A genetic association study of CSMD1 and CSMD2 with cognitive function. *Brain Behav. Immun.* 61 209–216. 10.1016/j.bbi.2016.11.026 27890662

[B4] Atwoli L. Stein D. J. Williams D. R. Mclaughlin K. A. Petukhova M. Kessler R. C. (2013). Trauma and posttraumatic stress disorder in South Africa: analysis from the South African Stress and Health Study. *BMC Psychiatry* 13:182. 10.1186/1471-244X-13-182 23819543 PMC3716970

[B5] Auton A. Abecasis G. R. Altshuler D. M. Durbin R. M. Abecasis G. R. Bentley D. R. (2015). A global reference for human genetic variation. *Nature* 526 68–74. 10.1038/nature15393 26432245 PMC4750478

[B6] Barbano A. C. van der Mei W. F. deRoon-Cassini T. A. Grauer E. Lowe S. R. Matsuoka Y. J. (2019). Differentiating PTSD from Anxiety and Depression: Lessons from the ICD-11 PTSD Diagnostic Criteria. *Depress. Anxiety* 36 490–498. 10.1002/da.22881 30681235 PMC6548615

[B7] Blauwendraat C. Nalls M. A. Singleton A. B. (2020). The genetic architecture of Parkinson’s disease. *Lancet Neurol.* 19 170–178. 10.1016/S1474-4422(19)30287-X 31521533 PMC8972299

[B8] Blevins C. A. Weathers F. W. Davis M. T. Witte T. K. Domino J. L. (2015). The Posttraumatic Stress Disorder Checklist for *DSM-5* (PCL-5): Development and Initial Psychometric Evaluation: Posttraumatic Stress Disorder Checklist for *DSM-5*. *J. Trauma Stress* 28 489–498. 10.1002/jts.22059 26606250

[B9] Chang C. C. Chow C. C. Tellier L. C. Vattikuti S. Purcell S. M. Lee J. J. (2015). Second-generation PLINK: rising to the challenge of larger and richer datasets. *GigaScience* 4:7. 10.1186/s13742-015-0047-8 25722852 PMC4342193

[B10] Choi S. W. O’Reilly P. F. (2019). PRSice-2: Polygenic Risk Score software for biobank-scale data. *GigaScience* 8 1–6. 10.1093/gigascience/giz082 PMC6629542 31307061

[B11] Christiansen D. M. Hansen M. (2015). Accounting for sex differences in PTSD: A multi-variable mediation model. *Eur. J. Psychotraumatol.* 6:26068. 10.3402/ejpt.v6.26068 25604705 PMC4300366

[B12] Daskalakis N. P. Rijal C. M. King C. Huckins L. M. Ressler K. J. (2018). Recent Genetics and Epigenetics Approaches to PTSD. *Curr. Psychiatry Rep.* 20:30. 10.1007/s11920-018-0898-7 29623448 PMC6486832

[B13] Delaneau O. Marchini J. Zagury J.-F. (2011). A linear complexity phasing method for thousands of genomes. *Nat. Methods* 9 179–181. 10.1038/nmeth.1785 22138821

[B14] Duncan L. E. Cooper B. N. Shen H. (2018a). Robust Findings From 25 Years of PTSD Genetics Research. *Curr. Psychiatry Rep.* 20:115. 10.1007/s11920-018-0980-1 30350223 PMC6209025

[B15] Duncan L. E. Ratanatharathorn A. Aiello A. E. Almli L. M. Amstadter A. B. Ashley-Koch A. E. (2018b). Largest GWAS of PTSD (N=20 070) yields genetic overlap with schizophrenia and sex differences in heritability. *Mol. Psychiatry* 23 666–673. 10.1038/mp.2017.77 28439101 PMC5696105

[B16] Durbin R. (2014). Efficient haplotype matching and storage using the positional Burrows-Wheeler transform (PBWT). *Bioinforma. Oxf. Engl.* 30 1266–1272. 10.1093/bioinformatics/btu014 24413527 PMC3998136

[B17] Fonkoue I. T. Marvar P. J. Norrholm S. Li Y. Kankam M. L. Jones T. N. (2020). Symptom Severity Impacts Sympathetic Dysregulation and Inflammation in Post-Traumatic Stress Disorder (PTSD). *Brain Behav. Immun.* 83 260–269. 10.1016/j.bbi.2019.10.021 31682970 PMC6906238

[B18] Gelernter J. Sun N. Polimanti R. Pietrzak R. Levey D. F. Bryois J. (2019). Genome-wide association study of post-traumatic stress disorder reexperiencing symptoms in >165,000 US veterans. *Nat. Neurosci.* 22 1394–1401. 10.1038/s41593-019-0447-7 31358989 PMC6953633

[B19] Gurka M. J. Filipp S. L. Musani S. K. Sims M. DeBoer M. D. (2018). Use of BMI as Marker of Adiposity in a Metabolic Syndrome Severity Score: Derivation and Validation in Predicting Long-term Disease Outcomes. *Metabolism* 83 68–74. 10.1016/j.metabol.2018.01.015 29410278 PMC5960618

[B20] Heuvel L. L. van den Stalder T. Plessis S. du Suliman S. (2020). Hair cortisol levels in posttraumatic stress disorder and metabolic syndrome. *Stress* 23 577–589. 10.1080/10253890.2020.1724949 32008379

[B21] Koomar T. Michaelson J. J. (2020). Genetic Intersections of Language and Neuropsychiatric Conditions. *Curr. Psychiatry Rep.* 22:4. 10.1007/s11920-019-1123-z 31953567

[B22] Lewis C. M. Vassos E. (2020). Polygenic risk scores: from research tools to clinical instruments. *Genome Med.* 12:44. 10.1186/s13073-020-00742-5 32423490 PMC7236300

[B23] Marees A. T. de Kluiver H. Stringer S. Vorspan F. Curis E. Marie−Claire C. (2018). A tutorial on conducting genome−wide association studies: Quality control and statistical analysis. *Int. J. Methods Psychiatr. Res.* 27:1608. 10.1002/mpr.1608 29484742 PMC6001694

[B24] McCarthy S. Das S. Kretzschmar W. Delaneau O. Wood A. R. Teumer A. (2016). A reference panel of 64,976 haplotypes for genotype imputation. *Nat. Genet.* 48 1279–1283. 10.1038/ng.3643 27548312 PMC5388176

[B25] McLaughlin K. A. Koenen K. C. Bromet E. J. Karam E. G. Liu H. Petukhova M. (2017). Childhood adversities and post-traumatic stress disorder: evidence for stress sensitisation in the World Mental Health Surveys. *Br. J. Psychiatry* 211 280–288. 10.1192/bjp.bp.116.197640 28935660 PMC5663970

[B26] Misganaw B. Guffanti G. Lori A. Abu-Amara D. Flory J. D. Sbpbc (2019). Polygenic risk associated with post-traumatic stress disorder onset and severity. *Transl. Psychiatry* 9:165. 10.1038/s41398-019-0497-3 31175274 PMC6555815

[B27] Ney L. J. Akhurst J. Bruno R. Laing P. A. Matthews A. Felmingham K. L. (2021). Dopamine, endocannabinoids and their interaction in fear extinction and negative affect in PTSD. *Prog. Neuropsychopharmacol. Biol. Psychiatry* 105 1–17. 10.1016/j.pnpbp.2020.110118 32991952

[B28] Nievergelt C. M. Maihofer A. X. Klengel T. Atkinson E. G. Chen C.-Y. Choi K. W. (2019). International meta-analysis of PTSD genome-wide association studies identifies sex- and ancestry-specific genetic risk loci. *Nat. Commun.* 10:12576–w. 10.1038/s41467-019-12576-w 31594949 PMC6783435

[B29] Nievergelt C. M. Maihofer A. X. Mustapic M. Yurgil K. A. Schork N. J. Miller M. W. (2015). Genomic predictors of combat stress vulnerability and resilience in U.S. Marines: A genome-wide association study across multiple ancestries implicates PRTFDC1 as a potential PTSD gene. *Psychoneuroendocrinology* 51 459–471. 10.1016/j.psyneuen.2014.10.017 25456346

[B30] Noda S. Sato S. Fukuda T. Tada N. Uchiyama Y. Tanaka K. (2020). Loss of Parkin contributes to mitochondrial turnover and dopaminergic neuronal loss in aged mice. *Neurobiol. Dis.* 136:104717. 10.1016/j.nbd.2019.104717 31846738

[B31] Nöthling J. Suliman S. Martin L. Simmons C. Seedat S. (2019). Differences in Abuse, Neglect, and Exposure to Community Violence in Adolescents With and Without PTSD and Depression. *J. Interpers. Violence* 34 4357–4383. 10.1177/0886260516674944 27777370

[B32] Patterson N. Price A. L. Reich D. (2006). Population Structure and Eigenanalysis. *PLoS Genet.* 2:e190. 10.1371/journal.pgen.0020190 17194218 PMC1713260

[B33] Penninx B. W. J. H. Lange S. M. M. (2018). Metabolic syndrome in psychiatric patients: overview, mechanisms, and implications. *Dialogues Clin. Neurosci.* 20 63–73. 10.31887/dcns.2018.20.1/bpenninx 29946213 PMC6016046

[B34] Polimanti R. Ratanatharathorn A. Maihofer A. X. Choi K. W. Stein M. B. Morey R. A. (2019). Association of Economic Status and Educational Attainment With Posttraumatic Stress Disorder. *JAMA Netw. Open* 2:3447. 10.1001/jamanetworkopen.2019.3447 31050786 PMC6503495

[B35] Price A. L. Patterson N. J. Plenge R. M. Weinblatt M. E. Shadick N. A. Reich D. (2006). Principal components analysis corrects for stratification in genome-wide association studies. *Nat. Genet.* 38 904–909. 10.1038/ng1847 16862161

[B36] R Core Team (2020). *R: A language and environment for statistical computing.* Vienna: R Foundation for Statistical Computing.

[B37] Rosenbaum S. Stubbs B. Ward P. B. Steel Z. Lederman O. Vancampfort D. (2015). The prevalence and risk of metabolic syndrome and its components among people with posttraumatic stress disorder: a systematic review and meta-analysis. *Metabolism* 64 926–933. 10.1016/j.metabol.2015.04.009 25982700

[B38] Sassone J. Serratto G. Valtorta F. Silani V. Passafaro M. Ciammola A. (2017). The synaptic function of parkin. *Brain J. Neurol.* 140 2265–2272. 10.1093/brain/awx006 28335015

[B39] Schurz H. Müller S. J. van Helden P. D. Tromp G. Hoal E. G. Kinnear C. J. (2019). Evaluating the Accuracy of Imputation Methods in a Five-Way Admixed Population. *Front. Genet.* 10:34. 10.3389/fgene.2019.00034 30804980 PMC6370942

[B40] Shalev A. Y. Gevonden M. Ratanatharathorn A. Laska E. van der Mei W. F. Qi W. (2019). Estimating the risk of PTSD in recent trauma survivors: results of the International Consortium to Predict PTSD (ICPP). *World Psychiatry* 18 77–87. 10.1002/wps.20608 30600620 PMC6313248

[B41] Shaltouki A. Sivapatham R. Pei Y. Gerencser A. A. Momčilović O. Rao M. S. (2015). Mitochondrial Alterations by PARKIN in Dopaminergic Neurons Using PARK2 Patient-Specific and PARK2 Knockout Isogenic iPSC Lines. *Stem Cell Rep.* 4 847–859. 10.1016/j.stemcr.2015.02.019 25843045 PMC4437475

[B42] Shen H. Gelaye B. Huang H. Rondon M. B. Sanchez S. Duncan L. E. (2020). Polygenic prediction and GWAS of depression, PTSD, and suicidal ideation/self-harm in a Peruvian cohort. *Neuropsychopharmacology* 45 1595–1602. 10.1038/s41386-020-0603-5 31926482 PMC7419528

[B43] Stephan A. H. Barres B. A. Stevens B. (2012). The Complement System: An Unexpected Role in Synaptic Pruning During Development and Disease. *Annu. Rev. Neurosci.* 35 369–389. 10.1146/annurev-neuro-061010-113810 22715882

[B44] Sullivan P. F. Agrawal A. Bulik C. M. Andreassen O. A. Børglum A. D. Breen G. (2018). Psychiatric Genomics: An Update and an Agenda. *Am. J. Psychiatry* 175 15–27. 10.1176/appi.ajp.2017.17030283 28969442 PMC5756100

[B45] The Schizophrenia Psychiatric Genome-Wide Association Study Consortium (2011). Genome-wide association study identifies five new schizophrenia loci. *Nat. Genet.* 43 969–976. 10.1038/ng.940 21926974 PMC3303194

[B46] Torrisi S. A. Leggio G. M. Drago F. Salomone S. (2019). Therapeutic Challenges of Post-traumatic Stress Disorder: Focus on the Dopaminergic System. *Front. Pharmacol.* 10:404. 10.3389/fphar.2019.00404 31057408 PMC6478703

[B47] Ueda S. Fujimoto S. Hiramoto K. Negishi M. Katoh H. (2008). Dock4 regulates dendritic development in hippocampal neurons. *J. Neurosci. Res.* 86 3052–3061. 10.1002/jnr.21763 18615735

[B48] Uren C. Hoal E. G. Möller M. (2020). Putting RFMix and ADMIXTURE to the test in a complex admixed population. *BMC Genet.* 21:845–843. 10.1186/s12863-020-00845-3 32264823 PMC7140372

[B49] Uren C. Kim M. Martin A. R. Bobo D. Gignoux C. R. van Helden P. D. (2016). Fine-Scale Human Population Structure in Southern Africa Reflects Ecogeographic Boundaries. *Genetics* 204 303–314. 10.1534/genetics.116.187369 27474727 PMC5012395

[B50] Vassos E. Di Forti M. Coleman J. Iyegbe C. Prata D. Euesden J. (2017). An Examination of Polygenic Score Risk Prediction in Individuals With First-Episode Psychosis. *Biol. Psychiatry* 81 470–477. 10.1016/j.biopsych.2016.06.028 27765268

[B51] Waszczuk M. A. Docherty A. R. Shabalin A. A. Miao J. Yang X. Kuan P.-F. (2020). Polygenic prediction of PTSD trajectories in 9/11 responders. *Psychol. Med.* 2020:S0033291720003839. 10.1017/S0033291720003839 33092657 PMC8186149

[B52] Watanabe K. Taskesen E. van Bochoven A. Posthuma D. (2017). Functional mapping and annotation of genetic associations with FUMA. *Nat. Commun.* 8:1826. 10.1038/s41467-017-01261-5 29184056 PMC5705698

[B53] Weathers F. W. Blake D. D. Schnurr P. Kaloupek D. G. Marx B. P. Keane T. (2015). *Clinician-Administered PTSD Scale for DSM-5 (CAPS-5).* Boston, MA: National Center for PTSD.

[B54] White D. L. Kunik M. E. Yu H. Lin H. L. Richardson P. A. Moore S. (2020). Post-Traumatic Stress Disorder is Associated with further Increased Parkinson’s Disease Risk in Veterans with Traumatic Brain Injury. *Ann. Neurol.* 88 33–41. 10.1002/ana.25726 32232880

[B55] WHO (2008). *The WHO STEPwise approach to noncommunicable disease risk factor surveillance (STEPS).* Geneva: World Health Organization. 10.2105/AJPH.2015.302962 PMC4695948 26696288

[B56] Wickham H. (2016). *ggplot2: Elegant Graphics for Data Analysis.* New York, NY: Springer-Verlag New York, 10.1007/978-0-387-98141-3

[B57] Willer C. J. Li Y. Abecasis G. R. (2010). METAL: fast and efficient meta-analysis of genomewide association scans. *Bioinformatics* 26, 2190–2191. 10.1093/bioinformatics/btq340 20616382 PMC2922887

[B58] Williams S. L. Williams D. R. Stein D. J. Seedat S. Jackson P. B. Moomal H. (2007). Multiple traumatic events and psychological distress: the South Africa stress and health study. *J. Trauma Stress* 20 845–855. 10.1002/jts.20252 17955545 PMC3269889

[B59] Wolf E. J. Miller D. R. Logue M. W. Sumner J. Stoop T. B. Leritz E. C. (2017). Contributions of polygenic risk for obesity to PTSD-related metabolic syndrome and cortical thickness. *Brain Behav. Immun.* 65 328–336. 10.1016/j.bbi.2017.06.001 28579519 PMC5537007

[B60] Woo H. J. Yu C. Kumar K. Reifman J. (2017). Large-scale interaction effects reveal missing heritability in schizophrenia, bipolar disorder and posttraumatic stress disorder. *Transl. Psychiatry* 7:e1089. 10.1038/tp.2017.61 28398343 PMC5416702

