# Supplementary material for: A Genome-Wide Association Study and Polygenic Risk Score Analysis of Posttraumatic Stress Disorder and Metabolic Syndrome in a South African Population
Source: Front Neurosci. 2021 Jun 10;15:677800. doi: 10.3389/fnins.2021.677800 (PMC8222611; doi:10.3389/fnins.2021.677800)
Supplement: Supplementary file 1 [file Data_Sheet_1.docx]

Supplementary Material

# Supplementary Figures


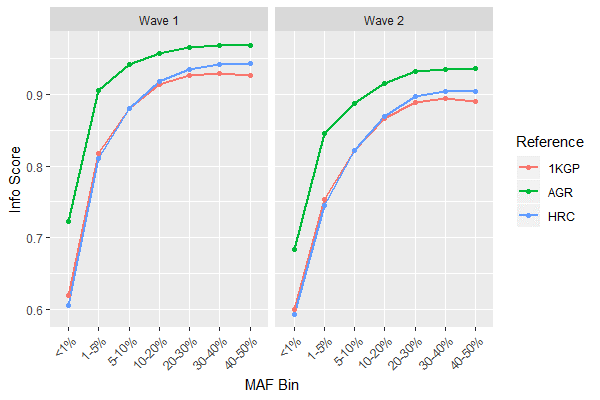


**Supplementary Figure 1.**: Mean INFO Scores (quality scores) per minor allele frequency (MAF) bin for chromosome 1 following imputation with three different reference panels [1KGP = 1000 Genomes Phase III (pink); AGR = African Genome Resource (green); HRC = Haplotype Reference Consortium (blue)] via the Sanger Imputation Server. Our results agree with Schurz et al., 2017, who also demonstrated that imputation using the AGR reference panel results in higher median quality scores compared to other available reference panels when conducting imputation in this study population.


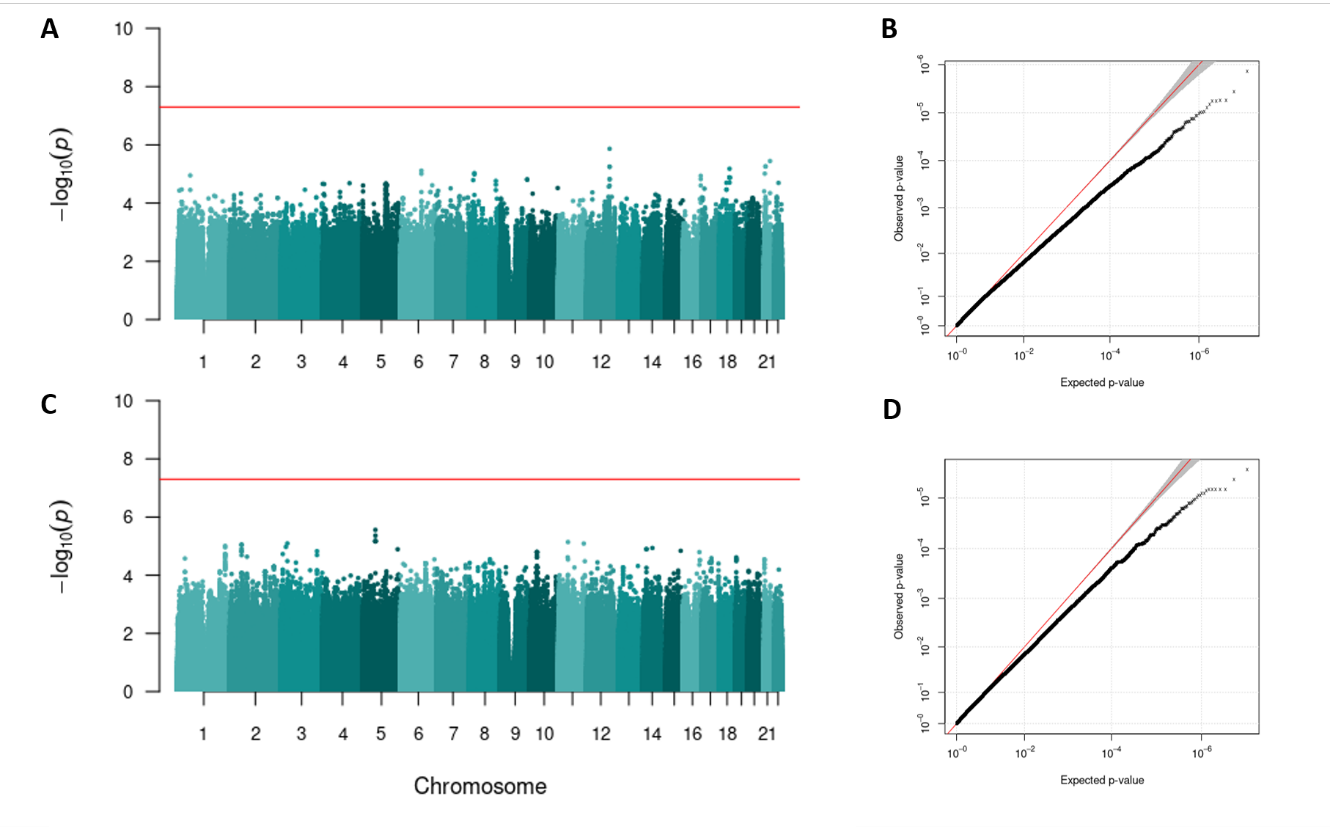


**Supplementary Figure 2.** (**A**) A Manhattan plot representing the GWAS conducted in Wave 1 participants (controls, n = 141; PTSD cases, n = 153, #SNPs = 11 927 194) and (**B**) the corresponding Q-Q plot of the expected vs. observed *p*-values (genomic inflation, λ = 1.015). Similarly, (**C**) illustrates the Manhattan plot from the GWAS conducted in Wave 2 participants (controls, n = 202; PTSD cases, n = 107, #SNPs = 10 300 047) and (**D**) the corresponding Q-Q plot of the expected vs. observed *p*-values (genomic inflation, λ = 1.002). No SNPs surpassed genome-wide significance threshold after Bonferroni correction (indicated by the horizontal red line, *p* < 5 x10^-8^).


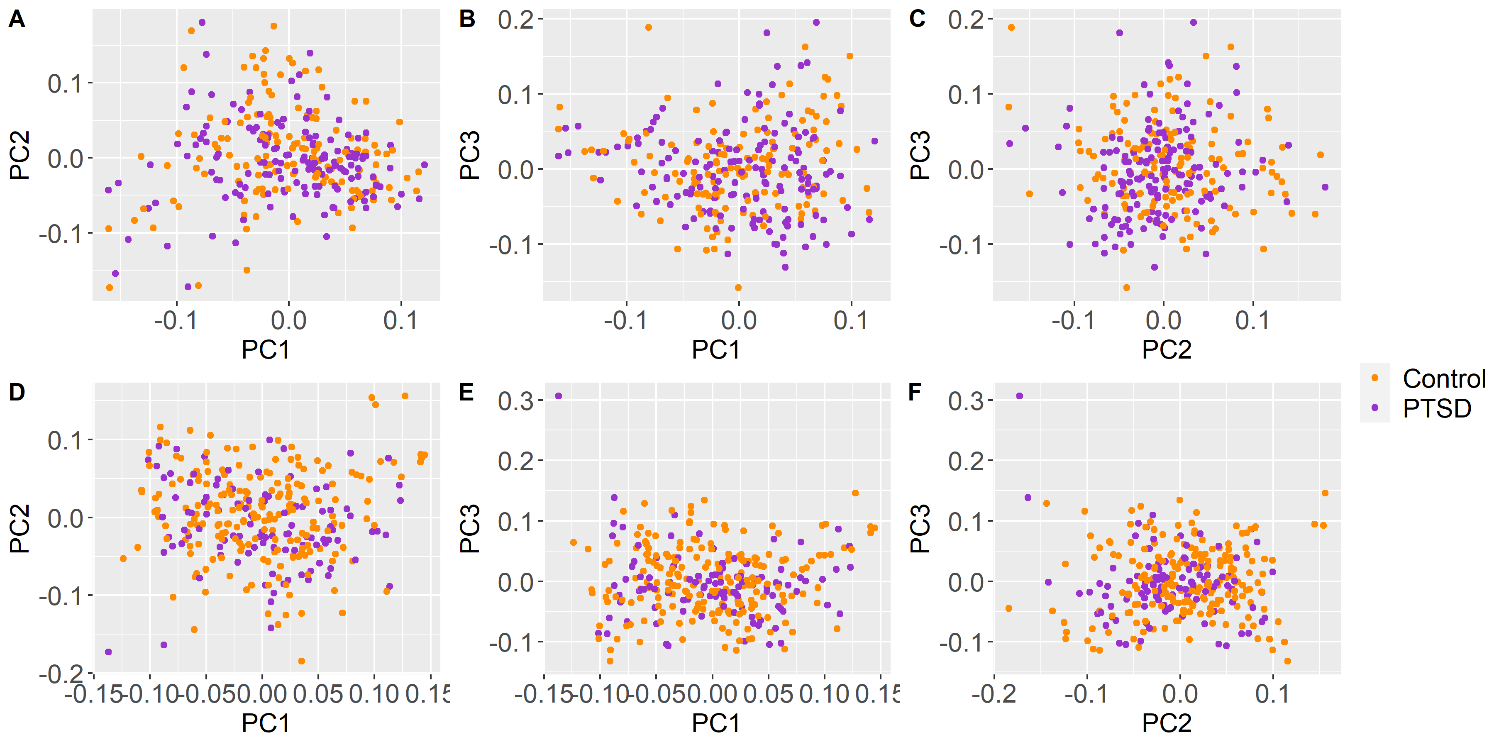


**Supplementary Figure 3.** Principal component analysis of controls and PTSD participants only. (**A-C**) Wave 1 and (**D-F**) Wave 2 controls and participants with PTSD plotted according to principal components (PC1 – 3). Controls and PTSD cases are evenly distributed across the principal components in both datasets.


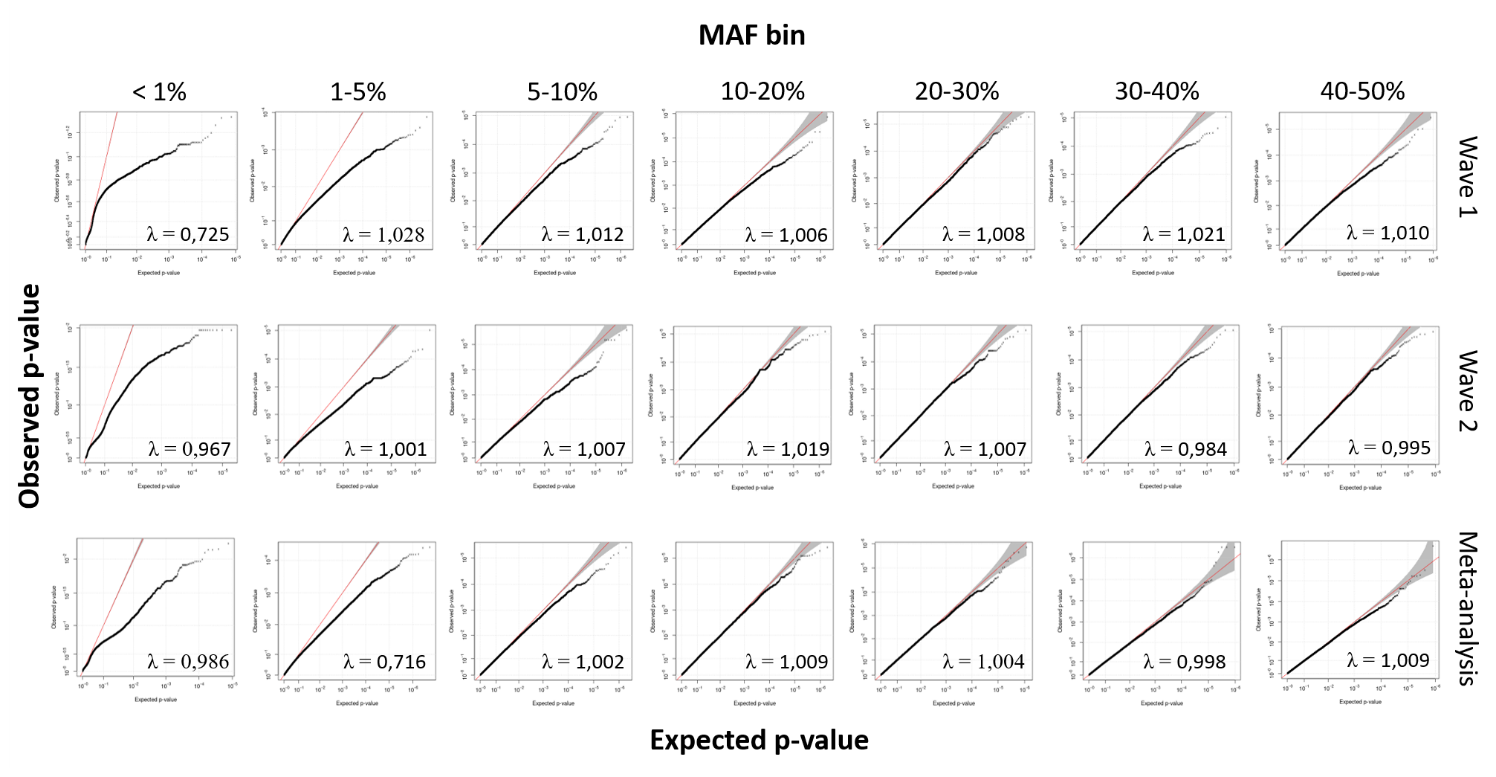


**Supplementary Figure 4:** Q-Q plots per minor allele frequency bin to investigate the source of the deflation observed in the association analyses.
